# Recent advances in edge illumination x-ray phase-contrast tomography

**DOI:** 10.1117/1.JMI.4.4.040901

**Published:** 2017-10-16

**Authors:** Anna Zamir, Charlotte Hagen, Paul C. Diemoz, Marco Endrizzi, Fabio Vittoria, Yujia Chen, Mark A. Anastasio, Alessandro Olivo

**Affiliations:** a University College London, Department of Medical Physics and Biomedical Engineering, London, United Kingdom; b Washington University in St. Louis, Department of Biomedical Engineering, St. Louis, Missouri, United States

**Keywords:** x-ray imaging, phase contrast, tomography, edge illumination

## Abstract

Edge illumination (EI) is an x-ray phase-contrast imaging technique, exploiting sensitivity to x-ray refraction to visualize features, which are often not detected by conventional absorption-based radiography. The method does not require a high degree of spatial coherence and is achromatic and, therefore, can be implemented with both synchrotron radiation and commercial x-ray tubes. Using different retrieval algorithms, information about an object’s attenuation, refraction, and scattering properties can be obtained. In recent years, a theoretical framework has been developed that enables EI computed tomography (CT) and, hence, three-dimensional imaging. This review provides a summary of these advances, covering the development of different image acquisition schemes, retrieval approaches, and applications. These developments constitute an integral part in the transformation of EI CT into a widely spread imaging tool for use in a range of fields.

## 1 Introduction

X-ray radiography has been in widespread use in the last century and has transformed numerous fields, including medicine and security scanning. While the relatively cheap technology offers many advantages for detecting features hidden inside opaque objects, it struggles to do so with weakly absorbing materials, or those with similar absorption properties. This is due to the fact that, in conventional radiography, image contrast is based on the difference in x-ray attenuation by different materials. However, apart from being attenuated, an x-ray beam passing through an object also undergoes phase changes. The extent of these effects is determined by the material’s complex refractive index, given by n(E)=1−δ(E)+iβ(E), where E is the x-ray energy and δ and β are linked to the phase and attenuation effects, respectively. Unlike conventional radiography, x-ray phase-contrast imaging (XPCi) methods use dedicated setups to detect phase effects, which can be considerably larger than attenuation effects. Different XPCi methods have been developed during the past two decades, the most prominent ones being propagation-based imaging (PBI), analyzer-based imaging (ABI), grating interferometry (GI), and edge illumination (EI).^1^^,^^2^ PBI is capable of detecting interference patterns caused by phase effects and has the simplest experimental setup as it requires no additional optical elements. It does however require spatially coherent radiation and a high-resolution detector. For these reasons, PBI is most suited for use in synchrotron radiation facilities;^3^ however, laboratory implementations using microfocal sources exist.^4^ ABI makes use of a crystal’s rocking curve to sense the refraction of x-rays^5^^,^^6^ and, therefore, uses only a narrow bandwidth of the x-ray beam and requires a highly stable setup. In practice, these two conditions mostly limit its use to a synchrotron environment,^7^ although lab-based implementations of ABI also exist.^8^^,^^9^ In GI, phase sensitivity is achieved by employing two gratings to create and detect the Talbot self-imaging effect.^10^ GI was first implemented using synchrotron radiation since the method relies on spatial coherence; however, GI can be made compatible for use with extended laboratory sources, provided that a third “source” grating is used.^11^ Similar to GI, the EI method can be used with both synchrotron and laboratory sources; however, it is noninterferometric and is based on a similar principle to ABI.^12^^–^^14^ In EI, x-ray refraction is detected using a slit (synchrotron implementation), or two masks (laboratory implementation), to create individual beamlets that impinge on an absorbing edge (synchrotron) or mask (laboratory). The EI method and its use as a computed tomography (CT) modality are the focus of this review, and hence, a detailed description of the working principle of EI is provided below.

## 2 Edge Illumination

**Fig. 1:**
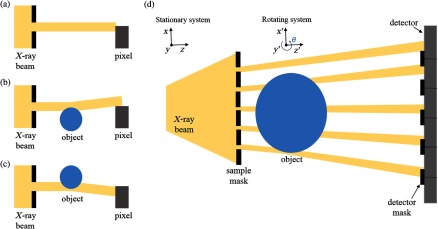
(a–c) Schematic diagrams of the synchrotron and (d) laboratory implementations of the EI system. An x-ray beam aligned with the edge of a pixel (a) is refracted away (b) or toward (c) the pixel after passing through an object. In the laboratory, (d) two masks are employed to replicate the EI condition over the entire field of view.

EI was first developed in the late 1990s using synchrotron radiation.^12^ The working principle of EI is demonstrated in Figs. 1(a)–1(c) in which a simplified schematic of the setup is shown. A narrow collimated beam is aligned with the edge of a detector pixel row or column, such that only part of the beam reaches the pixels. When an object is placed in the beam path, any refraction caused by the object will lead to a change in detected intensity, as it would deflect the beam toward or away from the pixel. In this example, upward refraction will lead to a decrease in measured intensity [see Fig. 1(b)] while refraction downward will increase the detected intensity [see Fig. 1(c)]. Practically, at a synchrotron, the incoming beam is collimated by a vertically narrow and horizontally long slit placed upstream of the object while an absorbing edge is placed in contact with the detector row to create the EI condition. Therefore, in this configuration, to obtain an image of the entire object, the latter must be scanned through the laminar beam. Since the method does not rely on interference effects, nor does it require monochromatic radiation, it is well suited for translation to a laboratory environment (using a polychromatic and extended source).^13^ To enable area imaging and avoid scanning the object (which would lead to impractical scan times when using a commercial source), the slit and edge are replaced with two masks, which replicate the EI condition for all detector rows (or columns), as shown in Fig. 1(d). The first mask (sample mask) is placed upstream of the object and divides the incoming beam into physically separated beamlets. The second mask (detector mask) is placed in contact with the detector and creates insensitive regions between pixels. The detector mask is fabricated with a period matching the detector pixel size while the sample-mask period is scaled down to account for the beam’s divergence. In this implementation, the entire object is captured in a single image with a spatial sampling rate given by the sample-mask period (demagnified pixel size). However, unlike most other XPCi methods, in EI the intrinsic spatial resolution is not limited by the pixel size and its upper limit is determined by the smaller of the sample-mask aperture and the projected source size, downscaled to the sample plane.^15^ To achieve this higher resolution, a process called “dithering” can be used, where multiple projections are acquired at different subpixel positions of the object and are later recombined to form a high-resolution projection.

**Fig. 2:**
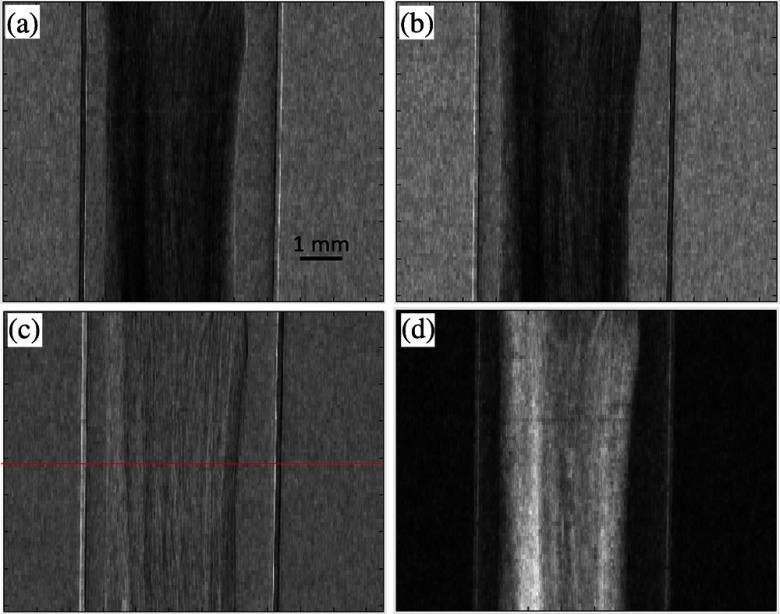
Unprocessed EI projections (a and b) of a rabbit’s esophagus, demonstrating a mixture of object attenuation and refraction contrast. By processing (a and b) together using a retrieval algorithm, these can be separated into (c) refraction and (d) attenuation projections. The red line in (c) corresponds to the position of the CT slice shown in Fig. 3(b). Note that although image quality is low in these projections (as each was acquired with low statistics), it is the combination of the statistics from all projections that ultimately leads to the high-image quality visible in Fig. 3(b).

Projections acquired with an EI system contain mixed information related to both the object’s attenuation and refraction properties. It has been shown that separate attenuation and refraction images can be obtained if two frames, acquired at different positions of the sample mask (where the beam is aligned with opposite edges of the pixel), are processed together.^16^^,^^17^ In the monochromatic case, this provides access to the following two quantities:

μ(x,y)=2k∫β(x,y,z)dz, (1)

$$\alpha (x,y)=k^{-1}\frac{\partial }{\partial x}\phi (x,y)=\frac{\partial }{\partial x}\int \delta (x,y,z)dz,$$ (2)

where μ is the linear attenuation coefficient, α is the refraction angle, ϕ is the phase function, and k is the wave number. In the polychromatic case, μ and α are also weighted over the x-ray spectrum.^18^ An example of the use of this retrieval algorithm is provided in Fig. 2, which shows projection images of a decellularized rabbit esophagus acquired using the lab-based EI system, for an application in the field of tissue engineering from an experiment described in Sec. 3.2. Mixed projections, corresponding to illuminating opposite edges of the pixel (and hence featuring an inverted differential phase signature), are shown in Figs. 2(a) and 2(b), whereas the refraction and attenuation projections resulting from the application of the retrieval algorithm are shown in Figs. 2(c) and 2(d), respectively.

Furthermore, if three frames are acquired and processed using a dedicated algorithm, a scattering image representing the object’s microscopic structure on a subpixel scale can be retrieved, in addition to the attenuation and refraction images.^19^^–^^21^ The object’s microscopic structure has the effect of broadening the beamlet. By defining $\sigma_{\phi }^{2}(x,y,z)dz$ as the localized broadening of the beamlet by an infinitesimal volume of the object of width dz, the retrieved scattering signal representing the total broadening of the beamlet is equal to^22^^–^^24^

$$\sigma_{f}^{2}=\int \sigma_{\phi }^{2}(x,y,z)dz.$$ (3)

These approaches, therefore, provide quantitative complementary information, essential for some applications. However, they typically require displacing the sample-mask multiple times, therefore making scans slower, especially in CT. To tackle cases where a single, high-quality image with high contrast is needed and the speed is more important than multimodality, a retrieval algorithm requiring only a single frame was developed for the case of homogeneous (or quasihomogeneous) objects.^25^

In general, the EI technique is extremely flexible as it can be adjusted for a range of applications, depending on specific requirements. Sensitivity to refraction on the scale of nanoradians has been observed using synchrotron radiation, and quantitative imaging has been demonstrated.^17^ On the other hand, the method is robust against environmental vibrations^26^ and achromatic,^27^ and it can be used with conventional extended x-ray tubes,^28^ making its translation to clinical or industrial laboratories possible. Note that, while the maximum acceptable source size depends on the specific setup parameters, for typical EI setups used so far, this is ∼100  μm,^28^ i.e., making the method compatible for use with mammography sources. Furthermore, the EI method is robust against increasing x-ray energy^29^ and can be scaled to larger fields of view.^30^ While the conventional EI setup is sensitive to refraction only in the direction perpendicular to the masks’ apertures, the system can be made sensitive to refraction in two directions if suitable masks are employed.^31^ EI can, therefore, be used for a vast range of applications, and indeed experiments were performed in diverse fields including security scanning, paleontology, tissue engineering, materials science, and mammography.^32^^–^^36^

## 3 Adaptation to Computed Tomography

The EI technique was first adapted to perform CT scans by Hagen et al.,^37^ by constructing separate sinograms from attenuation $\left(S_{\beta }\right)$ and refraction $\left(S_{\delta }\right)$ images, acquired at different rotation angles (θ) of the object

$$S_{\delta }(x,y;\theta )=k^{-1}\frac{\partial }{\partial x}\phi (x,y;\theta )=\frac{\partial }{\partial x}\int_{l(x,y;\theta ;s)}\delta (x^{\prime },y^{\prime },z^{\prime })ds,$$ (4)

$$S_{\beta }(x,y;\theta )=\mu (x,y;\theta )=2k\int_{l(x,y;\theta ;s)}\beta (x^{\prime },y^{\prime },z^{\prime })ds,$$ (5)

where $x^{\prime }=x\;\cos\;\theta -z\;\sin\;\theta$, $y^{\prime }=y$, and $z^{\prime }=x\;\sin\;\theta +z\;\cos\;\theta$ are the frame of reference of the object, and the line l(x,y;θ;s) describes the path of an x-ray hitting the detector at (x,y). Using Eqs. (4) and (5), tomographic maps of δ and β can be obtained with standard CT reconstruction algorithms, such as filtered back projection. The derivative appearing in Eq. (4) implies that a special filter function (the Hilbert filter^38^) must be used in the reconstruction (unless the signal is integrated beforehand).

### 3.1 Preliminary Results

The first CT scans using an EI system were carried out at the Elettra synchrotron facility in Trieste, Italy, where the feasibility of reconstructing quantitative δ and β maps was demonstrated for materials with a wide range of refractive index values.^37^ As an example of a low-attenuating object, CT reconstructions of a domestic wasp were presented, demonstrating the superiority of phase contrast over attenuation contrast by reporting a 9 times increase in the contrast-to-noise ratio. Furthermore, the theoretical background was developed and validated for retrieving “mixed” slices (i.e., ones formed as a linear combination of the phase and attenuation signals) for the case when the axis of rotation is parallel to the direction of phase sensitivity. These mixed slices, which have the appearance of edge-enhanced attenuation images, can be obtained from only one frame per projection and can aid in the identification of faint, weakly attenuating details.^37^^,^^39^

The first EI tomographic images obtained using a commercial laboratory x-ray tube were published shortly after.^40^ While employing a polychromatic spectrum, it was shown that quantitative tomograms of the complex refractive index can be obtained, if the concept of “effective energy”^18^ is adopted (see the supplementary material in Ref. 38). A previously developed model demonstrated that the latter depends on both system (source spectrum, masks attenuation, and detector energy response) and object (material and thickness) characteristics.^18^ More importantly, by reconstructing CT datasets of a biological sample, acquired with spatial sampling rates ranging from ideal sampling to the one given by the demagnified pixel size, it was shown that the phase signal strength is independent of the sampling rate. This observation, therefore, implies that EI CT has low-dose capabilities, since high image contrast can be maintained in datasets where no dithering is performed, thereby reducing the number of required images.

### 3.2 Tissue Engineering

**Fig. 3:**
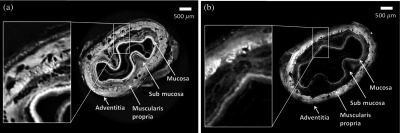
(a) Reconstructed slices of decellularized rabbit esophagi acquired using PBI with synchrotron radiation and (b) a laboratory-based EI setup. Adapted with permission from Macmillan Publishers Ltd.: Scientific Reports, Ref. 34, Copyright 2015.

A more recent collaboration was in the field of tissue engineering, where the possibility of using XPCi methods to visualize the microstructure of acellular scaffolds was investigated.^34^ By visualizing the scaffolds’ microstructure, different decellularization techniques can be evaluated, and the interaction between the scaffolds and the cells used to repopulate them prior to implantation can be studied. Unlike the currently used gold-standard techniques (histology and scanning electron microscopy),^41^ XPCi methods are nondestructive and, therefore, could be used prior to implantation, and possibly for *in vivo* monitoring.^34^ Different scaffolds were scanned using both ABI and PBI methods at the Elettra synchrotron (Trieste, Italy) and the European Synchrotron Radiation Facility (Grenoble, France), respectively, and the resulting image quality was sufficiently high to visualize all details of interest. A key finding resulting from this study was the observation that images obtained from scanning one of the scaffolds (a rabbit esophagus) with the laboratory EI system had comparable quality to the “gold-standard” phase contrast ones obtained with a PBI setup using synchrotron radiation.^34^ This can be appreciated by comparing the CT slices of rabbit esophagi shown in Fig. 3, noting that in both images, soft tissues are differentiated and the same anatomical structures can be identified and evaluated. These results suggest that EI CT could become a valuable tool for research, quality control, and monitoring in the field of tissue engineering.

### 3.3 Varying the Spatial Sampling Rate

**Fig. 4:**
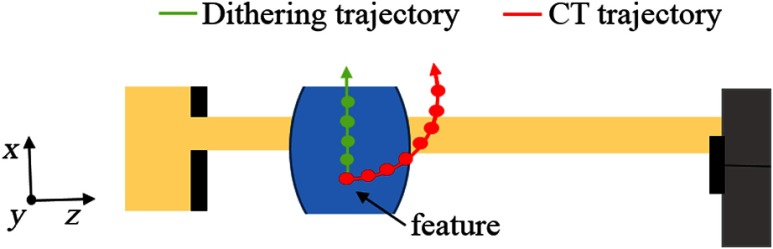
Increasing the spatial sampling rate: a subpixel feature in an object, which is not “seen” initially by the x-ray beamlet can be sampled either by dithering the object in the x-direction or by rotating the object.

In a previous study, the relationship between the spatial sampling rate (i.e., number of dithering steps) and the retrieval of quantitative phase information from EI images was investigated, for both planar and tomographic imaging modes.^42^ First, simulated and experimental data were generated for a wire phantom, using a very high sampling rate to guarantee that no undersampling occurs. Quantitative reference values of ϕ and δ were extracted from these data, for planar and tomographic imaging, respectively. The data were then continuously subsampled and ϕ and δ were extracted for each subsampled dataset. The optimal sampling rates for both planar and tomographic imaging were then determined, as the ones which resulted in the unambiguous retrieval of quantitative phase information (i.e., ones for which the retrieved quantities were independent of the sampling point position). The results of this analysis demonstrated that while a high sampling rate (i.e., a high number of dithering steps, in accordance with the Nyquist–Shannon theorem^43^^,^^44^) is required to retrieve unambiguous values of the phase shift in a planar image, a much reduced sampling rate can be used to retrieve quantitative tomograms of the refractive index decrement, δ. This result can be interpreted intuitively; the signal in a phase-retrieved EI projection is of differential nature (the quantity retrieved is proportional to the first derivative of the phase function), and hence, it contains high-frequency components, which require an appropriately high sampling rate to be effectively detected. However, in CT scans, the rotation of the object between projections leads to a similar outcome in terms of quantitativeness as the one obtained by increasing the number of dithering steps in a single projection (albeit at reduced spatial resolution). This concept is shown in Fig. 4 by comparing the dithering and CT trajectories of a feature in planar and tomographic imaging modes, respectively. This study provided a more rigorous confirmation of the previous observation according to which spatial resolution and quantitative phase information in EI CT are independent of each other. Hence, object-specific quantitative imaging can be performed, where the number of dithering steps is determined according to the required spatial resolution, therefore enabling the separate optimization of both dose control and scan duration.^42^

### 3.4 Retrieval Methods with Potential for Fast Imaging

#### 3.4.1 Reverse projection

**Fig. 5:**
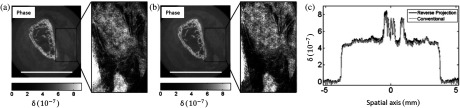
Phase tomograms of a chicken bone sample imaged with a conventional x-ray source and retrieved with the (a) reverse projection and (b) conventional EI retrieval algorithms. Scale bars indicate 5 mm. Quantitative values are compared in panel (c), where profiles extracted along the central horizontal lines in the respective tomograms are plotted together. Adapted with permission from IOP Publishing: Journal of Physics D: Applied Physics, Ref. 46, Copyright 2016.

An alternative retrieval method to the conventional two-image retrieval often used in EI CT^14^^,^^45^ was recently implemented.^46^ The algorithm removes the need for mask movement between projections and is based on the “reverse-projection” relation first developed for ABI to eliminate the equivalent requirement to reposition the crystal.^47^ Using this relation, for a given position of the sample mask, two projections separated by 180 deg can be processed together to extract separate refraction and attenuation information according to

$$\alpha (x,\theta )=F^{-1}[\frac{P(Mx,\theta )}{P(-Mx,\theta +\pi )}],$$ (6)

$$\mu (x,\theta )=-\ln\{\frac{P(Mx,\theta )}{C[x_{m}+z_{od}\alpha (x,\theta )/M]}\},$$ (7)

where P represents a projection acquired with the EI setup, M is the magnification between the sample mask and the detector, $x_{m}$ is the position of the sample-mask, $z_{od}$ is the object-to-detector distance, and y=const. is assumed. The illumination curve, C, describes the measured intensity as a function of the relative masks’ displacement in the absence of an object, and the function F can be calculated from the quantities M, $x_{m}$, $z_{od}$, and C.^46^ The reverse-projection relation holds for parallel beam geometries only, or ones that are approximately parallel (i.e., where the distortion introduced by the fan beam is smaller than the imaging system’s spatial resolution). Therefore, it can be applied to data acquired using synchrotron radiation, or using a conventional source, if the object is sufficiently small to satisfy the parallel beam approximation. Using the reverse-projection relation, a complete dataset is therefore acquired by rotating the object over 360 deg while keeping the sample-mask position fixed. Although there is no change in the total number of projections required with respect to the conventional two-image retrieval, the reverse-projection method simplifies the acquisition sequence by enabling a continuous rotation of the object. Moreover, the system is less prone to motor-induced misalignment, and scan times are reduced, in particular if a fast-readout detector is employed. The method was validated both quantitatively and qualitatively using EI CT datasets of a custom-built phantom and a biological sample, acquired in the laboratory.^46^ Figure 5 provides reconstructed phase maps of the biological sample, a chicken bone, using both the reverse-projection relation and the conventional retrieval, showing these lead to comparable results.

#### 3.4.2 Joint image reconstruction

Another retrieval method with the potential to reduce scan time is a single-shot technique, based on a joint reconstruction (JR) of the real and imaginary parts of the complex refractive index.^48^ This method overcomes the requirement for parallel beam geometries, which was imposed by the reverse-projection relation. Here, the retrieval of both δ and β is combined with the CT reconstruction step by using a gradient-based optimization method. An iterative algorithm, which requires only a single image per view, was developed. Using simulated data, it has been shown that both δ and β can be quantitatively retrieved either for datasets acquired over 360 deg with a constant position of the sample mask, or for ones acquired over at least 180 deg, where the position of the sample mask alternated between consecutive views. While the first option enables a continuous sample rotation, the second option requires less views and could be used for applications where rotating the object over 360 deg is not possible. CT reconstructions of a biological object imaged in the laboratory and retrieved with the JR algorithm were qualitatively and quantitatively comparable to the ones obtained with the conventional two-image retrieval algorithm.^48^

### 3.5 High-Resolution Scans

**Fig. 6:**
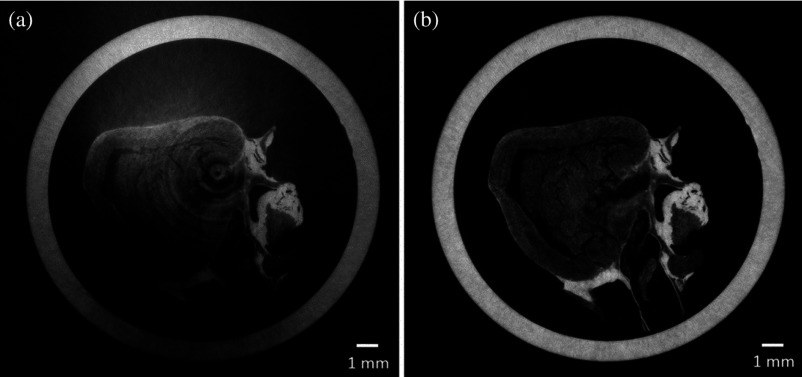
CT slices of a rat’s heart acquired at the laboratory and processed using (a) the conventional and (b) modified local phase retrieval algorithms. By tracking and correcting for the variation of system parameters over time, severe image artifacts are removed. Adapted with permission from Macmillan Publishers Ltd.: Scientific Reports, Ref. 49, Copyright 2016.

Although the development of fast, low-dose CT-scanning schemes has been the focus of many recent studies, certain applications (e.g., materials science and *ex vivo* medical research) are not necessarily limited by dose constraints and instead require high-resolution images, often with the additional requirement for quantitative retrieval of multiple contrast channels. Quantitative information of the object’s attenuation, refraction, and scattering properties can be obtained using a “local” retrieval algorithm, if three frames per projection are processed together, even in the case where the optical elements are locally imperfect or misaligned.^21^ However, high-resolution multimodal CT scans are inevitably long, as scan times are increased by a factor equal to the number of dithering steps used, and the retrieval of multiple contrasts requires the acquisition of three frames per dithering step. When these scans are performed in a laboratory, the system is exposed to environmental changes (e.g., vibrations and temperature changes), which affect the system’s stability. In a recent study, the way system parameters vary with time (in particular the lateral shift of the beamlets) was analyzed experimentally and modeled through a simulation, and was shown to lead to errors in the retrieved refraction signal.^49^ Since these errors typically vary with the projection angle, they lead to increased noise and artifacts. To account for these changes and remove the arising CT-image artifacts, a modification to the algorithm was introduced (modified local retrieval), in which a correction term for the beamlets shift is found through a fitting process, which uses information from a background region in each frame. Indeed, the use of the algorithm leads to the removal of major artifacts, as can be seen in Fig. 6 in which laboratory CT slices of a rat’s heart, processed with the conventional two-image retrieval and with the modified local algorithm are compared.^49^ Using this algorithm, high-resolution, accurate CT data can be reconstructed from long CT scans acquired in nonideal conditions (e.g., in clinical or industrial environments).

## 4 Discussion and Conclusion

The fundamental concepts of EI have been presented, along with the theoretical background for the method’s adaptation to a CT modality. Since the publication of the first CT results obtained with an EI system in 2014, there has been ongoing progress, aimed at advancing both hardware and software aspects of the technique, such that EI CT could be used for a wide range of applications and in different environments. Ultimately, the results of the studies reported here would lead to “object-specific” imaging: depending on the imaged object and its associated requirements and constraints, the flexibility of the method would allow striking the right balance between the delivered dose, spatial resolution, scan duration, and extracted quantitative information. Undoubtedly, the technique could be further improved and would benefit from additional thorough studies of its different components. In fact, as the EI technique developed, it has been the focus of several studies conducted by other groups,^50^^–^^52^ although so far, these were limited to planar EI imaging.

Currently, many efforts within our group are focused on developing strategies which will enable faster acquisitions with reduced dose. Progress in this particular topic will be a significant step toward establishing widespread use of EI CT in clinical laboratories. As discussed previously, two such approaches (reverse projection and JR) have already been demonstrated with laboratory CT data. Recently, Diemoz et al.^25^ developed another method, which requires only a single image per projection. This retrieval algorithm relies on the assumption of a homogeneous object and was successfully applied to planar data acquired using synchrotron radiation.

In addition, an alternative implementation of the method follows the “beam tracking” approach, where changes in the beam can be tracked using a high-resolution detector. In this implementation, the detector mask is removed while the sample-mask remains fixed throughout the scan, thereby leading to a simplified, faster acquisition. The method was successfully implemented at a synchrotron,^23^ and work is currently under way to implement it for CT scans in the laboratory.

In conclusion, EI CT is a promising technique, able to provide three-dimensional maps of an object’s refractive index properties, using both synchrotron radiation and conventional x-ray tubes. Considering the flexibility in its setup and acquisition parameters, we believe it could become a valuable nondestructive imaging modality used for applications in various fields, including but not limited to materials science, preclinical research, and regenerative medicine.
